# Advances in gut-lung axis research: clinical perspectives on pneumonia prevention and treatment

**DOI:** 10.3389/fimmu.2025.1576141

**Published:** 2025-04-22

**Authors:** Fang Ye, Linnan Li, Jiao Wang, Hongfeng Yang

**Affiliations:** Department of Critical Care Medicine, The Affiliated People’s Hospital of Jiangsu University, Zhenjiang, Jiangsu, China

**Keywords:** pulmonary infection, gut microbiota, gut-lung axis, immunity, probiotics

## Abstract

In recent years, the study of the interaction between gut microbiota and distant organs such as the heart, lungs, brain, and liver has become a hot topic in the field of gut microbiology. With a deeper understanding of its immune regulation and mechanisms of action, these findings have increasingly highlighted their guiding value in clinical practice. The gut is not only the largest digestive organ in the human body but also the habitat for most microorganisms. Imbalances in gut microbial communities have been associated with various lung diseases, such as allergic asthma and cystic fibrosis. Furthermore, gut microbial communities have significant impacts on metabolic function and immune responses. Their metabolites not only regulate gastrointestinal immune systems but may also affect distant organs such as the lungs and brain. As one of the most common types of respiratory system diseases worldwide, pulmonary infections have high morbidity and mortality rates. Pulmonary infections caused by immune dysfunction can lead to gastrointestinal problems like diarrhea, further resulting in imbalances within complex interactions that are associated with abnormal manifestations under disequilibrium conditions. Meanwhile, clinical interventions can significantly modulate the composition of gut microbiota, and alteration in gut microbiota may subsequently indicate susceptibility to pulmonary infections and even contribute to the prevention or regulation of their progression. This review delves into the interaction between gut microbiota and pulmonary infections, elucidating the latest advancements in gut-lung axis research and providing a fresh perspective for the treatment and prevention of pneumonia.

## 1 Introduction

Pneumonia is defined as a lung parenchymal infection that typically presents as localized inflammation in the lungs. However, in severe cases, persistent inflammatory response can lead to sepsis, organ failure, shock, and even death (1). Furthermore, there still exists a high incidence and mortality rate of pneumonia globally. According to statistics, approximately 102,000 people die from community-acquired pneumonia in the United States each year, with mortality rates being correlated with hospitalization time (2). The pathogens responsible for pulmonary infections are highly diverse, encompassing bacteria, viruses, fungi, and parasites. Among these, bacterial pneumonia is the most prevalent type, primarily caused by *Streptococcus pneumoniae*, *Haemophilus influenzae*, *Staphylococcus aureus*, *Escherichia coli, Klebsiella pneumoniae*, and *Pseudomonas aeruginosa*. The predominant pathogen of community-acquired pneumonia (CAP) is *Streptococcus pneumoniae*, whose capsular polysaccharide facilitates immune evasion through mechanisms such as complement inhibition and blocking Toll-like receptor ligand recognition. *Haemophilus influenzae* enhances its colonization and infectivity in the respiratory tract via virulence factors like pili and capsules. As a common opportunistic pathogen, *Staphylococcus aureus* produces toxins and enzymes that induce tissue damage and inflammatory responses (3–5). In hospital-acquired pneumonia (HAP) and ventilator-associated pneumonia (VAP), *Escherichia coli*, *Klebsiella pneumoniae*, *Acinetobacter baumannii*, and *Pseudomonas aeruginosa* are key pathogens (6), characterized by high levels of drug resistance and adaptability, which pose significant challenges to clinical management. Viral pneumonia represents another critical form of pulmonary infection, mainly caused by influenza viruses, respiratory syncytial virus, adenoviruses, and coronaviruses. The annual seasonal influenza epidemic may be influenced by environmental factors affecting the stability of the influenza virus (7). Respiratory syncytial virus is the predominant pathogen responsible for acute lower respiratory tract infections in infants, young children, and the elderly, often leading to bronchiolitis and pneumonia. Adenovirus binds to host cell surface receptors via fiber proteins, exhibiting specific tissue tropism and causing respiratory infections. Furthermore, adenovirus evades the host immune response by inhibiting interferon activity, apoptosis, and the expression of major histocompatibility complex class I molecules (8). Coronaviruses are enveloped RNA viruses with lipid layers on their outer surfaces; their spike proteins bind to host cell surface receptors, enabling cellular invasion and subsequent infection (9). Notably, the severe acute respiratory syndrome coronavirus 2 (SARS-CoV-2) has triggered the global public health crisis of the COVID-19 pandemic (10). Among these, the COVID-19 pandemic caused by Severe Acute Respiratory Syndrome Coronavirus 2 (SARS-CoV-2) has triggered a significant global public health crisis (11). Moreover, fungal pneumonia and pneumonia caused by opportunistic pathogens are also prevalent among patients with compromised immune function, such as pulmonary infections induced by *Cryptococcus*, *Aspergillus*, and *Pneumocystis jirovecii (* 12).

In fact, pulmonary infections including bacterial infections, fungal infections, and viral infections are all associated with dysbiosis of the lung microbiota. This differs from the perception of a sterile state in the lungs because in reality, the pulmonary microbial community is constantly undergoing dynamic migration and clearance (13). It’s worth noting that human microbiota is not limited to just the lungs; it also exists in various parts of the body such as the gastrointestinal tract (GI), skin, oral cavity,respiratory tract,and vagina (14). The majority of microorganisms reside in the gastrointestinal (GI) tract, where their total gene count surpasses that of human genes by several hundred times. A large and complex microbial ecosystem, known as the gut microbiota, exists within the human intestinal tract. This community is primarily composed of bacteria, archaea, fungi, and viruses, with bacteria playing a dominant role. Among the human intestinal bacteria, the key phyla include *Firmicutes (Bacillota)*, *Bacteroidetes (Bacteroidota)*, *Proteobacteria (Pseudomonadota)*, *Actinobacteria*, and *Fusobacteria (* 15). In a healthy human intestinal tract, *Firmicutes (Bacillota)* and *Bacteroidetes (Bacteroidota)* typically dominate, exhibiting genetic diversity in cytochrome P450 monooxygenase and ferredoxin genes. These microbial communities are not only essential for maintaining intestinal microecological balance and host health but may also exert diverse influences on human health (16). Therefore, the large number and abundance of gut microbiota may play an important role in influencing lung microbiota and subsequently affecting prognosis when patients have respiratory-related diseases. Certainly, existing evidence highlights the critical role of gut microbiota and its metabolites in sustaining human health. Abnormal alterations in the gut microbiota, referred to as dysbiosis, encompass changes in both composition and function. Dysbiosis is generally characterized by a reduction in beneficial bacteria, an expansion of potentially pathogenic bacteria, and a decline in microbial diversity. A variety of factors can induce this imbalance, including antibiotic usage, modifications in dietary patterns, infections, and immune system fluctuations. Dysbiosis has been closely associated with the onset, progression, and exacerbation of numerous diseases (17–19). However, when these steady states are disrupted, it not only affects GI tract but also impacts distal organs such as lungs, brain, and liver (20, 21). It is this close connection that brings together gut microbial communities and cutaneous immune systems into what best exemplifies the concept known as “gut-lung axis”. In this review article, we will focus on recent advancements in the field of “gut-lung axis”.

## 2 The causal relationship between gut microbiota and lung infections

There is a complex interaction between the gut microbiota and lung diseases. On one hand, lung diseases and respiratory infections can cause changes in the diversity and abundance of gut microbiota, leading to an ecological imbalance in the gut microbial community. Recent studies have shown that infection with novel coronavirus increases the number of pathogenic bacteria in patients’ intestines while reducing beneficial bacteria. In addition, coronavirus infection also reduces transcriptional activity in patients’ feces and is associated with longitudinal changes in fecal microbiota composition (22). Furthermore, analysis of infant fecal samples using 16S rRNA gene sequencing has revealed that respiratory syncytial virus infection affects gut microbial composition. Severe respiratory syncytial virus-infected patients (such as S247 cases) show increased relative abundance of bacterial taxa such as *Clostridium difficile, Moraxellaceae bacteria*, *Lactobacillus*, and *Actinobacteria* compared to healthy infants; meanwhile, there is a decrease in the quantity of *Moraxellaceae bacteria* in severe respiratory syncytial virus children (23). These alterations in microbial composition and function may disrupt intestinal immune balance and increase susceptibility to inflammatory-related issues within the intestine. On the other hand, disrupted gut microbiota can also impact the progression and prognosis of pulmonary problems; for example, there is an association between low-abundance specific bacterial groups at ages 4-7 years old and high risk for asthma development. Importantly, it should be noted that intestinal microecology can enhance immune responses within respiratory mucosa by regulating production components within small bodies which subsequently improves host resistance against viral infections or secondary bacterial infections (24–26). Additionally, intake of inhibitors can lead to a decrease in specific species within gut microbiota thereby affecting the occurrence of pulmonary diseases and allergic inflammation (27). All these findings confirm that the gastrointestinal tract and lungs are complex organ systems that together maintain homeostasis within the body.

### 2.1 Pneumonia has the potential to induce dysbiosis in the intestinal microbiota

**Figure 1:**
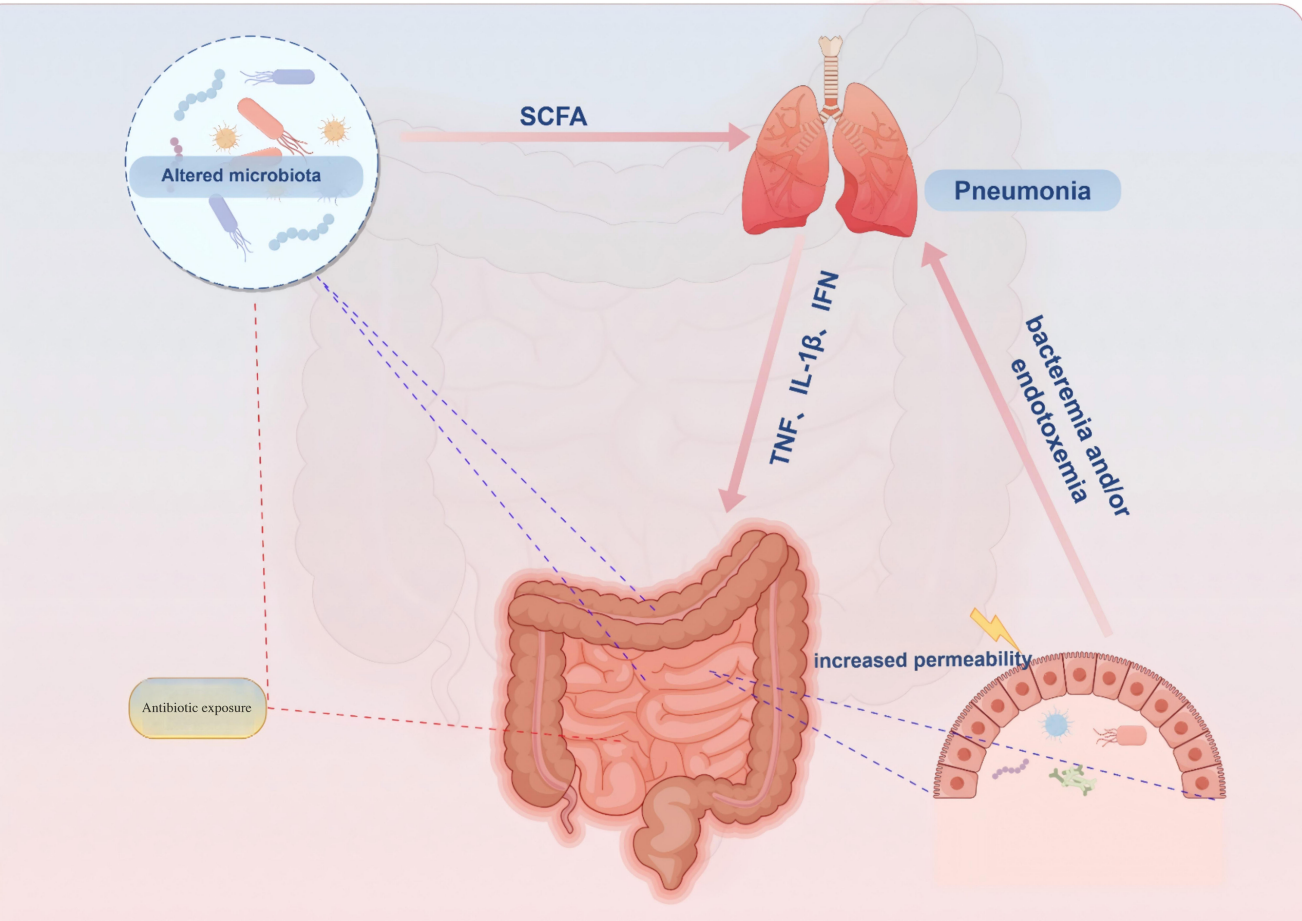
Proposed connections between the gut and lungs in pneumonia. Pneumonia is characterized by a pulmonary parenchymal infection, typically presenting as localized inflammation in the lungs and impaired epithelial barrier function. (1) Damaged and activated lung cells further stimulate the innate immune response through elevated proinflammatory mediators such as TNF-α, IFN-γ, and IL-1β. (2) These proinflammatory mediators can migrate to the gastrointestinal tract, partly via systemic circulation, exacerbating intestinal impairments including increased inflammatory immune infiltration and epithelial barrier damage. Additionally, antibiotic exposure and inflammatory immune cell infiltration in the gut can alter microbiota composition, leading to reduced abundance of health-promoting commensal bacteria that attenuate bacterial synthesis of immune-modulating compounds like SCFAs. (3) Impaired gut function not only increases production and entry of proinflammatory mediators into systemic circulation but also weakens protection against pathogens, thereby further worsening pneumonia symptoms by decreasing intestinal mucosa permeability while enhancing bacterial synthesis of immune-modulating compounds like SCFAs. SCFA = short-chain fatty acid.

A growing body of evidence suggests the involvement of the gastrointestinal system in the pathogenesis, progression, and prognosis of pneumonia patients undergoing treatment. Gastrointestinal symptoms, including diarrhea, are observed in 2%-20% of COVID-19 infected individuals (28). Moreover, COVID-19 patients with gastrointestinal symptoms (particularly diarrhea) exhibit reduced tryptophan synthesis (29). To investigate specific alterations in gut microbiota composition, a study employing deep sequencing of fecal samples from children with community-acquired pneumonia (CAP) revealed a decline in *Proteobacteria (Pseudomonadota)* and *Bacteroidetes (Bacteroidota)* as well as *Escherichia/Shigella*, *Prevotella*, *Clostridium sensu* stricto groups and *Enterobacteriaceae* among children aged 0-3 years old. Conversely, *Actinobacteria* and *Firmicutes (Bacillota)* along with *Bifidobacterium* and *Streptococcus* showed varying degrees of increase. In children aged 4-5 years old, there was a significant decrease in *Firmicutes (Bacillota)* while an increase was observed for *Actinobacteria* and *Streptomyces*. The changes at the genus level also exhibited significant differences (30). *Pseudomonas aeruginosa* infection can elicit a robust inflammatory response, thereby altering the diversity and composition of the gut microbiota. Specifically, the abundances of *Firmicutes (Bacillota)*, *Campylobacterota*, *Actinobacteriota*, and *Cyanobacteria* decreased, whereas the abundances of *Proteobacteria (Pseudomonadota)* and *Verrucomicrobiota* increased (31). Additionally, *Klebsiella pneumoniae* infection may modulate the host’s immune regulatory function by altering the metabolites of the gut microbiota, such as short-chain fatty acids (SCFAs) (32). Additionally, respiratory syncytial virus and influenza virus infections induced dysbiosis in mouse gut microbiota (33). In an acute lung injury model using mice, bacterial migration from lungs to intestines via blood circulation resulted in increased microbial load within the intestines leading to disruption of gut microbiota diversity (34). These findings highlight potential impacts of pulmonary infections on gut microbiota composition while potentially triggering gastrointestinal-related symptoms through immune regulatory pathways mediated by the ‘gut-lung’ axis. (**Figure 1**) However, whether gut microbiota dysbiosis is merely a “consequence” of pneumonia remains debated. For instance, a longitudinal study in stroke patients revealed that gut microbiota dysbiosis (e.g., reduced *Roseburia* levels) at admission independently predicted the risk of subsequent stroke-associated pneumonia (SAP). Moreover, the microbial dysbiosis index (MDI) was significantly correlated with both the severity and mortality of pneumonia (35).

### 2.2 Dysbiosis of the gut microbiota may impact pulmonary inflammation

Dysbiosis of the gut microbiota can alter the lung microbiota, exacerbate lung inflammation, and even serve as a negative prognostic factor for pulmonary infections. SARS-CoV-2 has been found to replicate in the gastrointestinal tract and potentially spread to other sites, such as the lungs, through its impact on inflammatory regulation and ACE2 expression in the gastrointestinal tract (36).Recent research has confirmed and clarified that tryptophan can enhance the expression of ACE2 and B0AT1, and activate the mTOR signaling pathway to repair intestinal injury (37).Research has indicated an association between specific gut microbial groups, such as *Enterobacteriaceae*, and community-acquired pneumonia (CAP) (30). Furthermore, an imbalance in the intestinal flora may influence brain-gut axis signaling by producing autoinducer-2 (AI-2), potentially contributing to the induction or exacerbation of post-stroke pneumonia (38). Conversely, certain gut microbial groups and their metabolites may play a protective role for the host. Reversing the restoration of the gut microbiota has been proven to mediate the de-colonization of Pseudomonas aeruginosa from the gastrointestinal tract, thus preventing its infection and spread (39). Short-chain fatty acids (SCFAs), commonly present in the gut, act as crucial immunoregulatory metabolites and have demonstrated protective effects in individuals with airway inflammation (40). Furthermore, depletion or loss of gut microbiota is believed to impact host immune response. A study by Schuijt et al. revealed that mice with depleted gut microbiota exhibited increased bacterial dissemination, inflammation, organ damage, and mortality compared to control mice. However, fecal microbiota transplantation (FMT) reversed diversity of gut microbiota and enhanced host defense against pneumonia (26). Those evidence implies that gut microbiota disorders might serve as an “antecedent” for certain types of pneumonia. It should be emphasized that the “causal effect” of gut microbiota on pulmonary infections requires validation through interventional studies. For example, fecal microbiota transplantation (FMT) in animal models has been shown to reverse antibiotic-induced microbiota dysbiosis, restore alveolar macrophage phagocytic function, and decrease pneumonia mortality rates (41). Additionally, research on pulmonary arterial hypertension patients indicates that gut microbiota metabolites (e.g., trimethylamine N-oxide [TMAO]) can indirectly exacerbate lung inflammation by promoting pulmonary vascular remodeling (42). These findings collectively support the notion that gut microbiota dysbiosis may act as a driving force behind lung diseases.

However, the causal relationship between gut microbiota dysbiosis and pulmonary infections could potentially be bidirectional: in immunosuppressed or chronically ill patients, microbiota dysregulation may enhance susceptibility to pneumonia by impairing mucosal immunity; conversely, acute pulmonary infections may secondarily disrupt intestinal homeostasis via inflammatory factor release, increased mucus secretion, or antibiotic use. Furthermore, future investigations should integrate multi-omics technologies and intervention experiments to comprehensively analyze this intricate interaction.

## 3 The distinct immunomodulatory characteristics of the gut microbiota in pneumonia

Increasing evidence supports the concept of a “common mucosal immune response,” highlighting the potential influence of gut microbial community and its metabolites on mucosal immunity, potentially extending to distant sites such as lung immune responses (43–45). Innate lymphoid cells (ILCs) are a population of antigen receptor-negative immune cells that reside on mucosal surfaces, playing a pivotal role in maintaining tissue homeostasis and eliciting immune responses. Despite their lower abundance in circulation, they exhibit high enrichment within mucosal barriers such as the intestines and airways. Within these mucosal tissues, ILCs play crucial roles in preserving internal equilibrium and participating in pathological alterations (46). In healthy individuals, ILCs actively participate in tissue repair processes. Over the past few years, research into the role of ILC2 cells in pneumonia and their interactions with the intestinal microbiota has gradually expanded. ILC2 cells regulate the inflammatory response in the lungs by secreting cytokines such as IL-5 and IL-13, which play pivotal roles in diseases like asthma and allergies (47). Studies have demonstrated that the activation and function of ILC2 cells are significantly modulated by the intestinal microbiota and its metabolites. It is noteworthy that activated intestinal ILC2 cells can employ lymphocyte function-associated antigen to egress from the intestine and migrate towards the lungs, thereby initiating airway inflammation (48). Furthermore, Huang et al.’s investigation has unveiled that type 2 innate lymphoid cells (ILC2s) can respond to inflammatory signals and subsequently migrate from the intestines to other organs including the lungs (49).These findings have offered a novel perspective on elucidating the role of ILC2s in pneumonia and their association with the intestinal microbiota. Likewise, Th17 and Treg cells are predominantly found on the mucosal surface, particularly in the lamina propria of the intestine. While Th17 cells induce autoimmune reactions and inflammation, Treg cells suppress these responses to maintain immune system homeostasis (50). However, pneumonia can disrupt the gut bacterial community, leading to an imbalance between Th17 and regulatory T (Treg) cells (51). Moreover, these commensal bacteria can enhance host resistance to bacterial pneumonia by upregulating IL-17A expression and promoting signaling pathways for granulocyte-macrophage colony-stimulating factor (GM-CSF) in lung epithelial cells (52).

Interestingly, the gut microbiota plays a crucial role in regulating the spatiotemporal dynamic balance of the mucosal immune system and exhibits distinct immune regulatory characteristics in bacterial, viral, and fungal pneumonia. The type of pathogenic microorganism directly influences the immune response pattern mediated by the gut microbiota.

### 3.1 The immunoregulatory characteristics of bacterial pneumonia: microbial flora metabolism and immune collaborative defense mechanisms

The immune regulation in bacterial pneumonia (e.g., infections caused by *Streptococcus pneumoniae* and *Pseudomonas aeruginosa*) is closely linked to the interaction between gut microbiota and host metabolism. Short-chain fatty acids (SCFAs), such as butyric acid, produced by the gut microbiota can circulate through the bloodstream to the lungs, where they enhance the bactericidal activity of alveolar macrophages and suppress excessive release of pro-inflammatory cytokines. For example, during *Pseudomonas aeruginosa* infection, butyrate modulates the maturation and effector functions of neutrophils, thereby influencing their recruitment and bactericidal capacity in the lungs and exerting complex regulation of the immune response (53). Furthermore, gut microbiota can upregulate the expression of Toll-like receptor 4 (TLR4) and activate the host’s defense mechanisms against Streptococcus pneumoniae infection (54). Additionally, gut microbiota enhances resistance to *Streptococcus pneumoniae* pneumonia and *Klebsiella pneumoniae* pneumonia via nucleotide-binding oligomerization domain protein 2 (NOD2) and granulocyte-macrophage colony-stimulating factor (GM-CSF) signaling pathways (52). Recent studies have demonstrated that abnormal proliferation of specific gut bacteria, such as *Eggerthella lenta*, impairs neutrophil function. These bacteria produce taurochenodeoxycholic acid (TUDCA), which inhibits the AMPK signaling pathway crucial for neutrophil energy metabolism, thereby reducing their phagocytic capacity against *Pseudomonas aeruginosa*. However, metformin, an AMPK activator, can reverse this defect, presenting a novel therapeutic approach for drug-resistant pneumonia (55). SFB can suppress the expression of pro-inflammatory genes in alveolar macrophages (AMs), thereby attenuating the inflammatory response and alleviating lung inflammation and injury. Moreover, SFB contributes to maintaining the homeostasis of AMs and prevents their depletion during viral infections, thus ensuring sustained protection of lung health and resistance to viral invasion (56). These findings suggest that the gut microbiota is involved in the immune regulation of bacterial pneumonia via multiple mechanisms, offering novel insights for the prevention and treatment of this condition.

### 3.2 The immunoregulatory characteristics of viral pneumonia: focus on the interferon pathway and immune cell migration

The immune regulation of viral pneumonia is intricately linked to the modulation of the interferon system by the gut microbiota. Deaminotyrosine (DAT), primarily produced by specific gut bacteria such as *Clostridium orbiscindens*, enhances type I interferon (IFN-I) signaling pathways, thereby promoting the expression of antiviral proteins like protein kinase R and MX1, which inhibit viral replication and dissemination. Animal studies have demonstrated that *Lactobacillus pentosus CCFM1227* augments the IFN-I response, leading to reduced lung inflammation and enhanced resistance to influenza virus infection (57, 58). In COVID-19 infections, dysbiosis of the gut microbiota decreases short-chain fatty acid (SCFA) levels, thereby weakening antiviral immunity in the lungs. Research has shown that severe COVID-19 patients exhibit a significant reduction in the abundance of butyrate-producing bacteria, including *Faecalibacterium*, *Roseburia*, and *Anaerostipes*. Supplementing with butyric acid can mitigate the “cytokine storm” induced by the virus (59, 60). Furthermore, the gut microbiota influences viral clearance and tissue repair by regulating the migration of Th17 cells and group 2 innate lymphoid cells (ILC2s) to the lungs. For instance, IL-17 plays a critical role in granulocyte colony-stimulating factor (G-CSF) expression and neutrophil homeostasis, while IL-5 secreted by ILC2s contributes to maintaining antiviral immune homeostasis (61, 62).

### 3.3 The immunoregulatory characteristics of fungal pneumonia: Th17 polarization and metabolite-mediated immune equilibrium

The intestinal flora plays a critical role in immune regulation during fungal pneumonia by modulating systemic immune responses and preserving the integrity of the intestinal barrier. Relevant studies demonstrate that the gut microbiota can regulate the polarization of CD4+ T cells during pulmonary fungal infection, thereby influencing lung immune defense mechanisms. For instance, vancomycin-treated drinking water significantly decreases the number of Th17 cells in the lungs during acute fungal infection, suggesting that the symbiotic effects of Gram-positive bacteria can modulate the expression of local antibacterial factors in the intestine and type 17 cytokines in the lungs. Additionally, the gut microbiota regulates the accumulation of Th17 cells in the lungs by affecting the expression of RegIIIγ (an IL-22-induced antibacterial protein specific to Gram-positive bacteria) and IL-22, thus impacting the immune response during fungal infection (63). Further research indicates that colonization with *segmented filamentous bacteria* (SFB) can induce the expansion of peripheral SFB-specific Th17 cells (64), which may influence the number of Th17 cells in the lungs. Moreover, metabolites of the gut microbiota also play a pivotal role. For example, short-chain fatty acids (SCFAs) enhance the production of bactericidal reactive oxygen species (ROS) by macrophages through histone deacetylase (HDAC) inhibition (65). Indole-3-carboxaldehyde, a metabolite produced by *Lactobacillus reuteri* during tryptophan catabolism, is involved in regulating host physiological processes and exhibits anti-inflammatory properties in various inflammatory diseases. The latest research indicates that I3A not only sustains lung epithelial barrier function through AhR receptor activation and IL-22 secretion stimulation but also inhibits HDAC5/6, resulting in the inactivation of the NF-κB/NLRP3 signaling pathway and the subsequent reduction of inflammatory factor expression (66). Additionally, studies have demonstrated that 5-hydroxytryptamine (5-HT), secreted by mast cells, plays a critical role in immune homeostasis during pathogen clearance and infection. This is achieved by promoting the host’s protective indoleamine-2,3-dioxygenase 1/kynurenine pathway while restricting the microflora-activated indole/aryl hydrocarbon receptor pathway. 5-HT exerts these effects via regulation of microbiota and signaling pathways in both lungs and intestines. Notably, supplementation with 5-HT has been shown to restore dysregulated tryptophan metabolism in mouse disease models, potentially offering a novel therapeutic strategy for pulmonary fungal infections (67).

### 3.4 Other pneumonias: unique pathogenic mechanisms of mycoplasma, chlamydia, and mixed infections

For atypical pathogens, such as *Mycoplasma pneumoniae*, or in cases of mixed infections, the regulatory mechanisms of the intestinal flora exhibit significant diversity. For instance, acetate and butyrate demonstrate potent anti-inflammatory effects on Mycoplasma pneumoniae-induced THP-1 cells. These short-chain fatty acids effectively suppress the activation of the NLRP3 inflammasome and Caspase-1, inhibit the nuclear translocation of NF-κB p65 protein, and consequently reduce the production and release of inflammatory factors. Moreover, acetate and butyrate decrease the levels of reactive oxygen species (ROS), enhance the secretion of anti-inflammatory cytokine IL-10 and immunoregulatory factor IFN-γ, thereby further mitigating the inflammatory response (68). Dysbiosis of the intestinal flora can also influence immune responses and the progression of *Chlamydia pneumoniae* infections. For example, using 16S rDNA sequencing technology, it has been demonstrated that children with *Chlamydia pneumoniae* exhibit impaired intestinal flora diversity and a marked reduction in Bifidobacterium abundance. Supplementation with *Bifidobacterium* can partially restore fecal microbial diversity, strengthen the intestinal barrier function, and exert a therapeutic effect on *mycoplasma* pneumonia (69, 70).

## 4 The role mechanism of intestinal microbiota and their metabolites in the regulation of pulmonary infection

### 4.1 Gut bacteria-derived Metabolites

Metabolites serve as the critical mediators of the interaction (crosstalk) between the gut microbiota and the human body. The gut microbiota generates a diverse array of metabolites, including volatile small molecules, lipids, proteins and peptides, sugars, secondary bile acids, terpenoids, biogenic amines, oligosaccharides, glycolipids, organic acids, and amino acids, among others (71). These metabolites are produced by the gut microbiota upon the absorption of specific substances and function as signaling molecules and reaction substrates during metabolic processes, playing a crucial role in the onset and progression of lung diseases, including lung infections. Furthermore, compared to microbial taxa, metabolites may be more suitable for research and intervention, as they not only reflect the functional attributes of microorganisms but also actively participate in the metabolic regulation between the host and microbiota (72). In subsequent sections, we will review several key gut microbial metabolites and investigate their potential promoting or inhibitory effects on lung infections.

#### 4.1.1 Short-chain fatty acids

Short-chain fatty acids (SCFAs) are pivotal metabolites generated by the intestinal microbiota via the fermentation of carbohydrates, particularly dietary fiber. These include acetic acid, propionic acid, and butyric acid. SCFAs exert a significant influence on both host intestinal health and systemic metabolism. Research has demonstrated that specific microbial groups are responsible for producing distinct types of SCFAs. For instance, members of the *Ruminococcaceae* and *Lachnospiraceae* families within the *Firmicutes (Bacillota)* are primary producers of butyric acid, whereas certain species in the *Bacteroidetes (Bacteroidota)* are crucial for the synthesis of acetic acid and propionic acid (73). Moreover, SCFAs produced in the gut not only achieve systemic distribution but also serve as an energy source and act as signaling molecules (74). Recent study has revealed that SCFAs can interact with G protein-coupled receptors to sense metabolites, thereby activating signaling pathways and inducing anti-inflammatory effects within the host’s immune response (75). Specifically, SCFAs generated by the intestinal microbiota through the fermentation of dietary fiber primarily enter systemic circulation via the portal vein and subsequently distribute to various organs, including the lungs (76). Within the lungs, SCFAs are absorbed and utilized by pulmonary immune cells, modulating the local immune response (77). Additionally, a study employed high-performance liquid chromatography (HPLC) to analyze cecal and serum samples from mice fed different dietary combinations. The findings revealed that increased intake of soluble dietary fiber significantly elevated SCFA levels in both cecal and serum samples. However, SCFAs were not detected in lung tissue samples (78). In contrast, another investigation identified SCFAs in the bronchoalveolar lavage fluid (BALF) of lung cancer patients, albeit at relatively low concentrations (79).

The presence of short-chain fatty acids (SCFAs) in the gut can effectively lower the pH of the intestinal lumen, thereby exerting inhibitory effects on the proliferation of pathogenic microorganisms. For instance, experimental evidence has demonstrated that acetate produced by *Bifidobacterium* is capable of suppressing pathogen growth within mouse intestines (80). Moreover, elevated levels of butyrate have been shown to enhance mucus production, leading to reduced bacterial adhesion and improved integrity of epithelial cells (80, 81). Oral supplementation with SCFAs can facilitate their entry into the bloodstream, potentially promoting the generation of protective regulatory T cells (Tregs) (82). Further investigations conducted on mice have revealed a correlation between butyrate production and an increase in Treg numbers. Additionally, *in vitro* experiments have elucidated the pivotal role played by butyrate in regulating forkhead box protein 3 (FOXP3), a lineage specification factor. As an inhibitor of histone deacetylases (HDACs), butyrate facilitates acetylation at FOXP3 gene promoter regions on histone H3 (83).SCFAs also exert an influence on dendritic cell development and inflammatory cytokine production, thereby modulating the function of intestinal macrophages (84). Moreover, SCFAs play critical roles in energy metabolism, which are essential for supporting B cell activation, differentiation, and antibody synthesis (85). Specifically, experimental studies have demonstrated that vancomycin administration in mice exacerbates allergic airway disease symptoms by altering the composition and abundance of gut microbiota, leading to reduced SCFA production. Conversely, supplementation with SCFAs via a high-fiber diet can effectively mitigate this adverse effect induced by vancomycin and alleviate disease severity (86, 87). Therefore, oral supplementation with SCFA supplements may also exhibit potential therapeutic efficacy in treating pulmonary diseases.

#### 4.1.2 Microbial tryptophan catabolites

As an essential aromatic amino acid, tryptophan primarily originates from dietary proteins. Its absorption predominantly occurs in the small intestine for protein synthesis. Concurrently, various bacteria in the colon can directly metabolize tryptophan into multiple derivatives, including indole, indole ethanol (IE), indole propionic acid (IPA), indole lactic acid (ILA), indole acetic acid (IAA), indole aldehyde (IAld), indole acrylic acid (IA), skatole, and tryptamine (88). Tryptophan and its derivatives are pivotal in the metabolism of the intestinal microbiota, modulating the host immune response and intestinal barrier function via diverse mechanisms. These metabolites also serve as critical signaling molecules in host-microbe cross-talk, preserving the integrity of the intestinal mucosal barrier and supporting the maintenance of intestinal and systemic homeostasis (89). For instance, IPA specifically binds to methionine adenosyltransferase 2A (MAT2A), enhancing S-adenosylmethionine synthesis and subsequently inducing DNA methylation of the deubiquitinating enzyme USP16. This process promotes ubiquitination and degradation of Toll-like receptor 4 (TLR4), thereby inhibiting the NF-κB signaling pathway and reducing IL-1β expression in M1 macrophages, ultimately alleviating lung inflammatory responses (90). Additionally, IPA functions as an allosteric inhibitor of TrpE by mimicking tryptophan, blocking the catalytic step of tryptophan synthesis mediated by TrpE, thus disrupting metabolic pathways in pathogens such as *Mycobacterium tuberculosis* and suppressing their proliferation (91). Animal studies demonstrate that influenza infection significantly alters the intestinal tryptophan metabolic pathway, characterized by a marked reduction in blood IPA levels; however, IPA supplementation effectively decreases viral load and mitigates both local (lung) and systemic inflammatory responses (92). IAA activates the aryl hydrocarbon receptor (AhR) to regulate pulmonary immune responses and reduce inflammation (93). ILA inhibits the polarization of mouse Th17 cells *in vitro* and stimulates the secretion of IL-22 by lamina propria lymphocytes through activation of the aryl hydrocarbon receptor (AhR), thereby promoting the proliferation of intestinal epithelial cells and repairing damaged intestinal mucosa (94). This regulatory mechanism not only aids in controlling infections but also effectively mitigates excessive inflammatory responses, thus protecting lung tissue. Recent studies indicate that in OVA-induced mouse asthma models, tryptophan metabolites such as KYN, ILA, I3C, and IAA can modulate the Th17/Treg balance via AHR activation, significantly reducing inflammation and improving survival rates. Specifically, KYN primarily restores the diversity of the gut microbiota, whereas ILA and I3C markedly enhance the proliferation of bacteria associated with tryptophan metabolism, such as *Akkermansia* and *Ruminococcus_1*. While KYN focuses on restoring overall microbial diversity, ILA and I3C specifically increase the relative abundance of these bacteria (95). Additionally, tryptophan metabolites regulate the interaction between intestinal commensal bacteria and the intestinal barrier by activating nuclear receptors, including the aryl hydrocarbon receptor (AhR) and the pregnane X receptor (PXR), further maintaining intestinal homeostasis and host health. Decreased levels of these metabolites may result in immune dysregulation. These metabolites can function independently or interact synergistically to jointly regulate intestinal homeostasis, offering novel therapeutic strategies for intestinal diseases.

#### 4.1.3 Conjugated linoleic acid

Conjugated Linoleic Acid (CLA) is a type of polyunsaturated fatty acid that is metabolized by intestinal flora and primarily derived from dietary linoleic acid. Certain gut microbiota, such as *Bifidobacterium* and *Lactobacillus*, can convert linoleic acid into CLA, thereby modulating host metabolism and immune function (96, 97). CLA plays an immunoregulatory role in the gut-lung axis by regulating the activity of immune cells and inflammatory responses. For instance, CLA can effectively promote the expression of IL-35 in macrophages via the Gαq/11-mediated STAT1/4 signaling pathway (98). Notably, a meta-analysis of the effects of CLA on inflammatory factors in adults indicates that CLA reduces levels of pro-inflammatory cytokines (e.g., TNF-α and IL-6), while slightly increasing C-reactive protein (CRP) levels (99). This suggests that CLA may exhibit complex pleiotropic effects in inflammatory responses, demonstrating both anti-inflammatory properties and potential pro-inflammatory characteristics depending on the isomer type or dose. Therefore, further studies are required to elucidate its specific mechanisms and biological significance. This regulatory effect aids in maintaining immune homeostasis in the lungs and mitigating inflammatory responses. Additionally, CLA enhances the immune system’s anti-infective capacity by regulating macrophage activity and differentiation and inhibiting excessive reactive oxygen species (ROS) synthesis (100). The mechanism by which conjugated linoleic acid (CLA) regulates immune responses is both intricate and multifaceted. CLA can directly modulate the activity of immune cells while also indirectly influencing immune regulation through enhancement of intestinal barrier function and modulation of the gut microbiota composition. The Jiang research group successfully established a neonatal rat model of necrotizing enterocolitis (NEC) by simulating the intestinal microenvironment of preterm infants using a multi-factorial induction approach that combined hypoxia, hyperosmolar formula feeding, and lipopolysaccharide (LPS) stimulation. Experimental findings revealed that the CLA intervention group significantly suppressed the overexpression of pro-inflammatory cytokines (IL-6, TNF-α) by activating the peroxisome proliferator-activated receptor γ (PPARγ) signaling pathway. Simultaneously, it restored the expression levels of intestinal tight junction proteins (e.g., occludin and claudin-1), thereby effectively enhancing intestinal barrier function (101). This may reduce the translocation of pathogens and toxins across the intestinal barrier, potentially lowering the risk of lung infections. Furthermore, under inflammatory conditions, CLA may influence host metabolism and immune status by reshaping the gut microbiota composition (increasing the abundance of beneficial bacteria such as *Peptostreptococcaceae* and reducing harmful bacteria like *Desulfovibrionaceae*). Huang et al. stimulated TNF-α-induced human bronchial epithelial (BEAS-2B) cells with varying doses of cis-9, trans-11-conjugated linoleic acid (c9,t11-CLA) and demonstrated that c9,t11-CLA significantly inhibited the secretion of inflammatory cytokines (e.g., IL-6, IL-8, MCP-1, and CCL5) and reduced the expression level of intercellular adhesion molecule 1 (ICAM-1), thereby attenuating monocyte adhesion to inflamed bronchial epithelial cells (102). Subsequent investigations revealed that c9,t11-CLA effectively suppresses the expression of pro-inflammatory factors, chemokines, and ICAM-1 by inhibiting NF-κB transcriptional regulation and dampening MAPK signaling pathway activity, thus exhibiting potent anti-inflammatory properties. Moreover, in a prospective, randomized, double-blind, placebo-controlled trial, conjugated linoleic acid (CLA) as a dietary supplement has been shown to offer potential benefits for overweight patients with mild asthma. The study indicated that CLA not only improves airway hyperresponsiveness (AhR) but also reduces body mass index (BMI), highlighting its critical role in modulating immune responses and inflammatory processes (103). Additionally, CLA may support the improvement of chronic obstructive pulmonary disease (COPD) conditions by suppressing oxidative stress production and regulating serum matrix metalloproteinase-9 (MMP-9) levels. Despite the limited number of studies on the specific mechanisms of CLA in pulmonary infections, its status as an intestinal flora metabolite suggests that CLA may indirectly influence lung health by modulating gut microbiota composition and function. Therefore, future research should further explore the potential application value of CLA in pulmonary infections and inflammatory diseases, which could facilitate the development of novel CLA-based therapeutic strategies.

#### 4.1.4 Secondary bile acids

Bile acids are synthesized from cholesterol in the liver via oxidative pathways. The primary bile acids include cholic acid (CA) and chenodeoxycholic acid (CDCA). These bile acids conjugate with taurine or glycine to form conjugated bile acids. The intestinal microbiota metabolically modifies bile acids through diverse pathways, specifically involving the following mechanisms (1): Deconjugation: Bile salt hydrolase (BSH), secreted by gut microbes such as *Firmicutes (Bacillota)*, *Bacteroidetes (Bacteroidota)*, *Actinobacteria*, *Proteobacteria (Pseudomonadota)*, and *archaea*, hydrolyzes conjugated bile acids into free bile acids (2). Dehydroxylation: Certain gut bacteria, particularly *Clostridium* species, convert primary bile acids into secondary bile acids, such as deoxycholic acid (DCA) and lithocholic acid (LCA), via 7α/β-dehydroxylase activity (3). Oxidation and epimerization: Gut microbes further diversify bile acid structures through oxidation and epimerization reactions (4). Sulfation and esterification: Specific gut bacteria alter the solubility and toxicity of bile acids through sulfation and esterification, thereby influencing their excretion (104). As key metabolites of the gut microbiota, bile acids enhance the solubilization of dietary lipids in the small intestine by forming micelles, thus promoting lipid absorption and excretion. Moreover, bile acids function as signaling molecules that interact with nuclear receptors (e.g., FXR) and G protein-coupled receptors (e.g., TGR5). Through these interactions, they regulate bile acid synthesis and metabolism, modulate lipid and glucose homeostasis, and participate in immune response regulation (105). Compared with the healthy control group, the intestinal metabolite profile of patients with community-acquired pneumonia (CAP) showed significant changes. Among them, the level of arachidonic acid significantly increased, while secondary bile acids (such as deoxycholic acid, lithocholic acid, and ursodeoxycholic acid) significantly decreased. This phenomenon suggests that the alteration of bile acid metabolism may play an important role in regulating the pulmonary inflammatory response (106), providing a potential direction for anti-inflammatory treatment strategies targeting bile acids. *In vitro* studies have shown that bile acids can regulate the expression level of angiotensin-converting enzyme 2 (ACE2) by activating the farnesoid X receptor (FXR), thereby influencing the pulmonary inflammatory response (107). Additionally, in the acute lung injury model, significantly upregulating the expression of FXR can inhibit the phosphorylation of p38 MAPK and NF-κB p65, reduce the release of inflammatory factors, and thereby protect lung tissue (108). At the same time, in the lipopolysaccharide-induced acute lung injury (ALI) model in rats, ursodeoxycholic acid (UDCA) and chenodeoxycholic acid (CDCA) not only enhanced the activity of antioxidant enzymes (including superoxide dismutase [SOD], catalase [CAT], and glutathione [GSH]), effectively alleviated oxidative stress, but also inhibited the inflammatory response by downregulating the expression of the pro-inflammatory transcription factor NF-κB. Specifically, it manifested as a reduction in the production of pro-inflammatory cytokines TNF-α, IL-6, and IL-1β, which play a key role in the pathogenesis of ALI. Furthermore, UDCA and CDCA can also upregulate the expression of aquaporin AQP1 and AQP5, improve the fluid balance in the lungs, and alleviate pulmonary edema. The aforementioned findings demonstrate that UDCA and CDCA exert multi-faceted protective effects on ALI by modulating oxidative stress, inflammatory responses, and fluid balance (109). Furthermore, bile acids exhibit concentration-dependent regulation of respiratory tract antimicrobial peptides and innate immune responses. At low concentrations, bile acids can enhance the expression of antimicrobial peptides in the respiratory epithelium and strengthen innate immunity via activation of relevant signaling pathways (e.g., TGR5-ERK1/2). Conversely, at higher concentrations, this promoting effect is suppressed, indicating a dual regulatory role of bile acids on respiratory immune responses depending on their concentration (110). In summary, bile acids hold potential therapeutic significance for maintaining pulmonary immune homeostasis.

#### 4.1.5 Polyamines

Polyamines are a class of metabolites produced by the intestinal microbiota, primarily comprising putrescine, spermidine, and spermine. These metabolites play crucial roles in maintaining intestinal and lung health. Polyamines are predominantly generated via the decomposition of dietary proteins and amino acids by the intestinal microbiota. For example, certain intestinal bacteria, such as *Bifidobacterium*, *Clostridium*, *Enterococcus*, *Lactobacillus*, and *Pediococcus*, can convert amino acids into polyamines through decarboxylation (111). Polyamines are essential for maintaining intestinal barrier function, regulating immune responses, and promoting cell proliferation (112). The composition and diversity of the intestinal flora significantly influence polyamine production. Studies have demonstrated that *Bacteroides* plays a pivotal role in the intestinal microbiota, and changes in its relative abundance are closely associated with polyamine biosynthesis levels. For instance, research conducted by Li’s team revealed that *Bacteroides* is the predominant genus involved in polyamine biosynthesis. In patients with inflammatory bowel disease (IBD), alterations in the relative abundance of Bacteroides are linked to variations in polyamine levels. Through single-bacteria stable isotope tracing analysis and computational simulations, the study further elucidated that *Bacteroides* synthesizes polyamines via specific metabolic pathways, underscoring the critical role of the intestinal microbiota in polyamine metabolism (113). Polyamines influence lung health through the gut-lung axis. Under pulmonary inflammatory conditions, the levels of different polyamine types may exhibit inconsistent changes. Research has shown that cadaverine levels increase in asthma patients, whereas spermidine levels decrease. Spermine and spermidine can modulate LPS-induced NF-κB activation, and oral administration of polyamines alleviates airway inflammation (114). Furthermore, polyamine metabolism plays a pivotal role in the growth, virulence, and stress response of *Streptococcus pneumoniae*, serving as a critical factor in its pathogenesis (115). Recent studies have demonstrated that spermidine effectively inhibits the phosphorylation of signal transducer and activator of transcription 1 (STAT1) by upregulating protein tyrosine phosphatase non-receptor 2 (PTPN2), thereby promoting macrophage polarization toward the anti-inflammatory M2 phenotype and suppressing excessive inflammatory responses. In a methicillin-resistant *Staphylococcus aureus* (MRSA) infection model, mice treated with spermidine exhibited significantly improved survival rates and enhanced bacterial clearance, providing robust experimental evidence for spermidine’s potential application in treating infectious diseases and laying the groundwork for its clinical use as an immunomodulator (116). Future research should focus on elucidating the precise mechanisms by which polyamines influence lung diseases. By unraveling these mechanisms, more effective therapeutic strategies can be developed, offering novel insights and approaches for the clinical management of lung diseases.

#### 4.1.6 Trimethylamine-N-oxide

Trimethylamine (TMA) is produced through the metabolism of specific dietary compounds by the gut microbiota and subsequently absorbed into the bloodstream (117). It primarily arises from the catabolism of nutritional substrates such as phosphatidylcholine, choline, carnitine, betaine, dimethylglycine, and ergothioneine by the colonic microbiota (118). Once absorbed, TMA can be oxidized to trimethylamine N-oxide (TMAO) by hepatic flavin monooxygenases (FMO1 and FMO3). Additionally, a portion of TMA may undergo further degradation in the colon, yielding methylamine, dimethylamine (DMA), and ammonia. The production of TMA is modulated by the balance and diversity of the gut microbiota. The higher ratio of *Firmicutes (Bacillota)* to *Bacteroidetes (Bacteroidota)* is associated with a greater response to the dietary precursors of TMAO, suggesting that TMAO production may be influenced by individual variations in the intestinal microbiota. An imbalance in the intestinal flora induces vascular damage by activating macrophages, which promotes atherosclerosis progression, thereby increasing the risk of coronary heart disease (119). In the rat model of diabetic nephropathy, dietary supplementation with trimethylamine-N-oxide (TMAO) and choline was found to significantly enhance the activation of the NLRP3 inflammasome. This, in turn, promoted the increased secretion of IL-1β and IL-18, triggering renal inflammatory responses and exacerbating oxidative stress through the overproduction of reactive oxygen species (ROS) (120). Futhermore, TMAO exacerbates TNF-α-mediated renal inflammatory responses by promoting the release of cytokines, chemokines, inflammatory mediators, and growth factors from renal fibroblasts (121). Also, study has demonstrated that in the presence of TMAO, the addition of nobiletin can inhibit vascular oxidative stress and suppress the expression and phosphorylation of ERK/NF-κB. Consequently, this results in an upregulation of Bcl-2 mRNA expression, a downregulation of Bax mRNA expression, and a reduction in downstream inflammatory factors, ultimately leading to the inhibition of inflammation (122).

#### 4.1.7 Polysaccharide A

Polysaccharide A (PSA) is a carbohydrate antigen secreted by the symbiotic bacterium *Bacteroides fragilis*. Prior studies have demonstrated that T cells exposed to PSA can synergize with tissue-resident FoxP3+ regulatory T cells (Tregs) to promote the secretion of the inhibitory cytokine IL-10 by Tregs, thereby effectively suppressing inflammatory cascade reactions and safeguarding lung health (123). Furthermore, a high-protein diet markedly elevates the level of the phenylalanine derivative PAGln produced by the gut microbiota, exacerbating colitis symptoms in mouse models. Notably, *Proteobacteria (Pseudomonadota)* harboring the phenylpyruvate decarboxylase (PPDC) gene are regarded as the primary contributors to PAGln production in patients with Crohn’s disease (CD) (124). Based on current research findings, both PSA and PAGln play potentially critical roles in the immune regulation of infectious diseases. Specifically, PSA can suppress inflammatory responses via its interaction with T cells, whereas the metabolic levels of PAGln may be influenced by alterations in the composition of the gut microbiota and exhibit pro-inflammatory effects in inflammatory bowel disease (IBD). Future investigations should delve deeper into the immune regulatory mechanisms of PSA and PAGln in specific infection models (e.g., pneumonia) and their functional interplay in the context of host-microbiota interactions. These research outcomes will offer crucial theoretical foundations and novel research avenues for the development of personalized medical strategies and the refinement of immunotherapies.

To sum up, the intestinal microbiota and its metabolites play a pivotal role in the immune regulation of various types of pneumonia. They modulate immune cell functions and inflammatory responses via multiple pathways, thereby influencing pathogen clearance and lung tissue repair. However, current research still has certain limitations. First, most studies concentrate on specific pathogens or single immune pathways, with limited exploration of mixed infections or multi-pathway synergistic effects. Second, clinical studies are relatively scarce; the majority of research relies on animal models or *in vitro* experiments, and the translatability of their findings to humans requires further validation. Future research directions should encompass the following aspects: conducting more clinical trials to assess the therapeutic efficacy and safety of interventions targeting the gut microbiota (e.g., probiotics, fecal microbiota transplantation) for various types of pneumonia; investigating the regulatory mechanisms of the gut microbiota in mixed infections and multi-pathogen scenarios to elucidate its immunomodulatory roles in complex infections; and considering factors such as individual variability and microbial diversity that may influence research outcomes, thereby enhancing the precision and reproducibility of studies.

### 4.2 Microbial translocation

**Figure 2:**
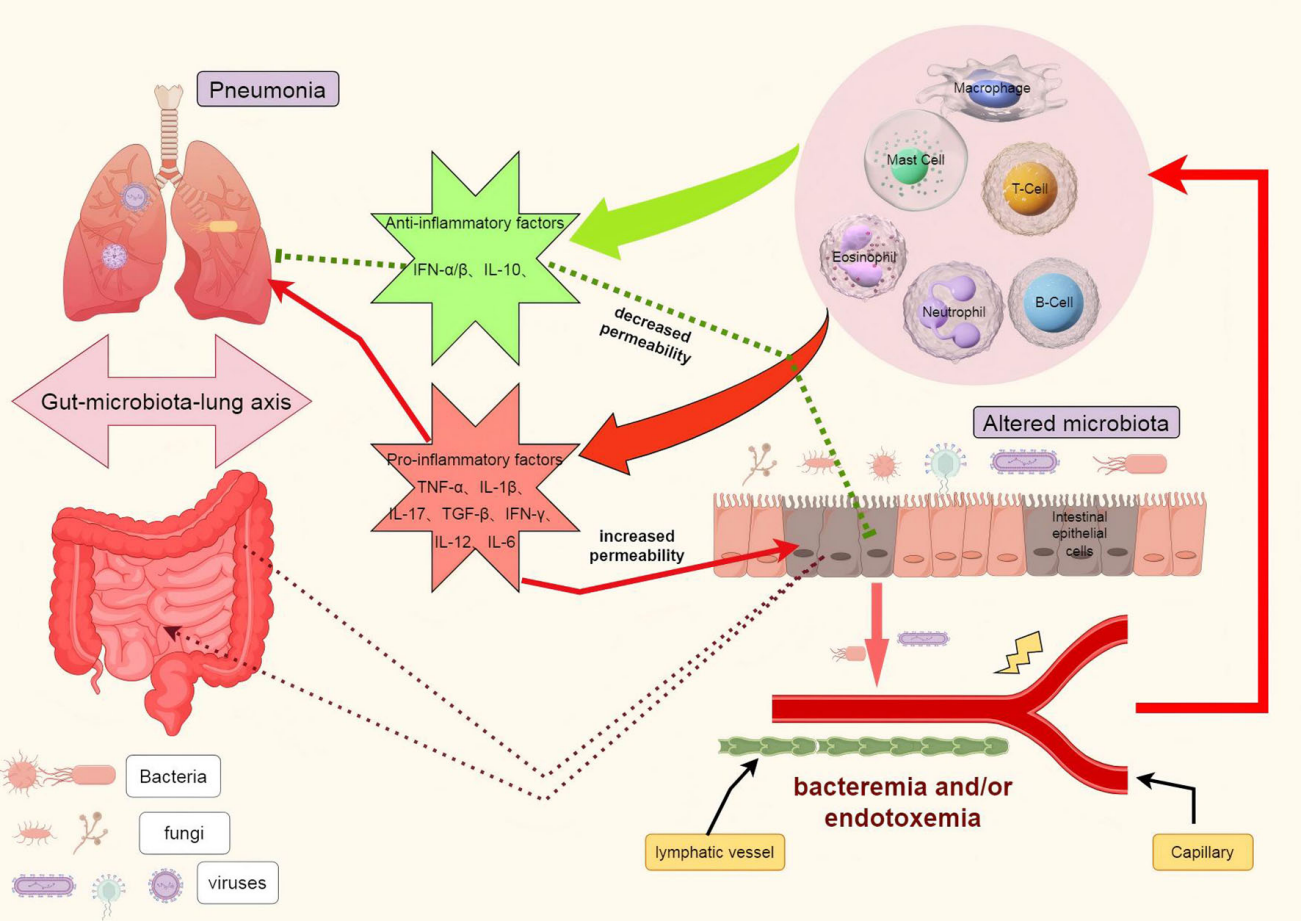
The role of bacterial translocation in the gut-lung axis. In the context of illness, the disruption of the intestinal barrier facilitates the translocation of pathogenic bacteria, leading to dysbiosis in the intestinal flora. These bacteria can be transported to the lungs via the lymphatic system and/or bloodstream, where they activate immune cells such as macrophages, neutrophils, T cells, and B cells. This activation results in the release of cytokines including IL-1, IL-6, IL-10, IL-12, TNFα, and IFN, which may either exacerbate or alleviate pulmonary infections and acute lung injury. Furthermore, these inflammatory factors can also reciprocally influence the intestinal barrier, potentially worsening or mitigating its damage. TNF-α tumor necrosis factor α, IL interleukin, IFN-γ interferon γ.

The maintenance of intestinal barrier function relies on the equilibrium of gut microbiota, integrity of the mucosa, and normal functioning of the immune system. Once one or more of these protective mechanisms are compromised, bacteria or their products such as endotoxins (lipopolysaccharides, LPS) may breach the intestinal epithelium and disseminate to mesenteric lymph nodes and even distant organs like liver and lungs. This phenomenon is known as bacterial translocation (125). During bacterial translocation, bacteria initially adhere to intestinal epithelial cells, followed by rupture of these cells’ membranes allowing bacteria to infiltrate and reach the basement membrane. Subsequently, through drainage from the intestine, bacteria can migrate to mesenteric lymph nodes where they have potential for further dissemination to other organs and tissues. Studies have revealed an enrichment of gut bacteria in lung microbiota among patients with acute respiratory distress syndrome (ARDS), providing novel evidence for a pivotal role played by bacterial translocation in the gut-lung axis (126). Furthermore, bacterial translocation is closely associated with alterations in intestinal barrier function, bacterial overgrowth, and impairment of host defense mechanisms. (**Figure 2**).

The healthy intestinal mucosal mechanical barrier consists of intact epithelial cells and the cell-to-cell junction complex, which includes tight junction proteins (TJ proteins). The function of the intestinal mucosal barrier is closely associated with the regulation of TJ protein levels. Indeed, the permeability of the intestinal mucosa is considered a crucial factor in innate defense, and disruption of barrier function can lead to excessive activation of immune cells (127). Inflammatory cytokines such as tumor necrosis factor-alpha (TNF-α), interferon-gamma (IFN-γ), interleukin-1 beta (IL-1β), and IL-12 disrupt the TJ barrier in the intestine during inflammation, thereby increasing TJ permeability (128). Moreover, IL-6 modulates claudin-2 protein expression through IL-6Rα-associated signaling transducers and gp130 signaling pathway, consequently enhancing TJ permeability in intestinal epithelium (129). Additionally, anti-inflammatory cytokine IL-10 plays a critical role in maintaining intestinal homeostasis by preserving intestinal barrier integrity; decreased levels of IL-10 and increased levels of IL-17 may exacerbate inflammation in a specific manner (130, 131).

In the gastrointestinal tract, a well-balanced symbiotic microbial community can confer health benefits to the host, while reduced microbial diversity may serve as an early indicator of certain pathological conditions. Both bacterial and viral-induced lung infections have the potential to disrupt the respiratory microbiota, leading to dysbiosis in the host gut microbiome along with compromised mucosal barrier and immune function, thereby promoting the development of lung infections. Deng et al.’s study unveiled that Ge-Gen-Qin-Lian decoction plays a protective role in safeguarding mice against influenza virus-induced pneumonia by partially improving the gut microbiota composition (such as increasing *Akkermansia*, *Desulfovibrio*, and *Lactobacillus* abundance while reducing *Escherichia coli*), inhibiting NOD/RIP2/NF-κB signaling pathway activation, and modulating expression of inflammatory cytokines in mesenteric lymph nodes (mLNs) and serum (132).

Gut-associated lymphoid tissue (GALT), being the largest immune organ in the human body, constitutes 25% of mucosal cell mass. In a thymus-deficient mouse model, spontaneous transfer of certain naturally occurring bacteria (e.g., *Escherichia coli*) to mesenteric lymph nodes (MLN), spleen, and liver has been observed; however, this phenomenon was not evident in heterozygous mice or nude mice receiving thymus transplantation (125). Subsequent investigations have demonstrated that T cell depletion leads to bacterial accumulation in rat MLN, while alcohol consumption and burns exacerbate bacterial alterations in rat MLN and increase the risk of bacterial translocation (133). Clearly, there exists a discernible correlation between host immune status and bacterial translocation.

Under healthy conditions, the host immune system efficiently prevents bacterial translocation by preserving intestinal barrier integrity, modulating microbiota balance, and regulating inflammatory responses. Gut-associated lymphoid tissue (GALT) plays a pivotal role in this process. It not only monitors pathogens within the intestine but also maintains intestinal homeostasis by generating immune cells and factors. For example, T cells and B cells in GALT can recognize and eliminate translocated bacteria while simultaneously producing anti-inflammatory cytokines such as IL-10 to sustain intestinal immune tolerance (134, 135).Moreover, innate immune cells recognize pathogen-associated molecular patterns via pattern recognition receptors (e.g., Toll-like receptors) and activate signaling pathways like NF-κB, thereby modulating the intensity and duration of the immune response (136). Conversely, under pathological conditions, the host immune system’s ability to regulate bacterial translocation becomes compromised. For instance, patients with chronic intestinal inflammatory diseases, such as inflammatory bowel disease (IBD), often exhibit impaired intestinal barrier function and increased mucosal permeability, which facilitates bacterial translocation (137).

## 5 Key mediators in the immune regulation of the gut-lung axis: PAMPs and DAMPs

Pattern recognition receptors (PRRs) serve as key mediators in the immune regulation of the gut-lung axis by recognizing pathogen-associated molecular patterns (PAMPs) and damage-associated molecular patterns (DAMPs) (138). PAMPs are molecular structures derived from pathogens, such as lipopolysaccharides or endotoxins from bacterial cell walls, which are recognized by intracellular pattern recognition receptors, including Toll-like receptors. Through the modulation of transcriptional initiation and signal cascade reactions, PRRs facilitate the elimination of pathogens (139). In contrast, the main difference of damage-associated molecular patterns is that they recognize endogenous molecular structures associated with damaged cells. Microbe-related molecular patterns can activate host pattern recognition receptors (such as NOD1 and NOD2), thereby activating downstream signaling pathways (140). Mutations in NOD proteins are associated with various inflammatory diseases affecting nuclear factor κB (NF-κB) activity, which is a key signaling pathway involved in apoptosis, inflammation, and immune response (141, 142).

TLRs are canonical transmembrane PRRs expressed on the surface of intestinal epithelial cells and alveolar macrophages, which specifically recognize pathogen-associated molecular components. For instance, upon recognizing LPS from Gram-negative bacteria, TLR4 activates NF-κB and MAPK signaling via the MyD88-dependent pathway, inducing the release of pro-inflammatory cytokines such as IL-6 and TNF-αto clear pathogens in the intestine or lungs (143, 144);Meanwhile, the TLR2/6 heterodimer recognizes bacterial toxins, promoting Th17 cell differentiation to enhance immune responses (145). It is worth noting that there exists a synergistic effect in the activation of Toll-like receptors (TLRs) in both the intestine and lungs. Specifically, TLR4 and TLR5 in the intestine recognize specific components of the gut microbiota (GM) and contribute to the immune defense mechanism against the influenza virus during this process (146). In contrast, intracellular NOD-like receptors (NLRs), such as NOD1/NOD2, primarily sense bacterial peptidoglycan fragments (e.g., iE-DAP and MDP). Their signaling activates the NF-κB and pro-inflammatory body pathways via RIP2 kinase, thereby regulating the production of antibacterial peptides in intestinal Paneth cells and the repair of the lung epithelial barrier (147, 148). Studies have demonstrated that mutations in the NOD2 gene are associated with susceptibility to Crohn’s disease and severe pneumonia, potentially due to lung immune imbalance caused by intestinal flora dysregulation (149, 150). Furthermore, recent research has uncovered that the gut microbiota regulates the metabolic reprogramming of alveolar macrophages through the NLRP6 inflammasome, enhancing neutrophil extracellular traps (NETs) and influencing bacterial clearance rates in the lungs (151, 152). This discovery offers a novel perspective on the immune interaction mediated by pattern recognition receptors (PRRs) within the gut-lung axis.

To sum up, PRRs function as a “two-way regulator” in the collaborative defense of intestinal and pulmonary immunity through tissue-specific expression and signal cross-talk. Nevertheless, their excessive activation may also result in immunopathological damage, indicating that targeting the PRR pathway necessitates careful consideration of both spatiotemporal specificity and the homeostasis of the host microenvironment.

## 6 The influence of clinical treatment intervention

### 6.1 Antibiotics

The physiological structure of the mucosal surfaces in the upper respiratory tract and intestines harbors distinct microbial communities. In a state of health, these microbial communities play a crucial role in preventing potential pathogen colonization and regulating immune responses to mitigate lung infections. However, the administration of antibiotics and intensive care procedures can disrupt the equilibrium of these microbial communities, compromising their protective functions and consequently elevating the susceptibility to lung infections.

Antibiotics may affect the nutritional metabolism of the gut by altering the abundance, diversity, and evenness of gut microbiota, reducing levels of short-chain fatty acids, regulating solute secretion and absorption, and decreasing secondary bile acid production. This can subsequently promote the occurrence of antibiotic-associated diarrhea (153). Research suggests that long-term exposure to antibiotics can damage the quantity and diversity of distal gut microbiota, even in the absence of clinically related gastrointestinal symptoms. The use of azithromycin significantly reduces gut microbial diversity in the short term, particularly leading to a significant decrease in *Actinobacteria*. Although there is some recovery over time, this dysbiosis may persist for up to three years (154). In contrast, disruption caused by macrolide antibiotics has a shorter duration but can still last up to two years. Similarly, studies on mouse models have revealed that after using neomycin sulfate, clearance of sensitive intestinal bacteria increases susceptibility to influenza virus infection in lungs (24, 155), and antibiotic use may lead to dysbiosis in gut microbiota communities which increases mortality risk due to bacterial infections (26). Conversely, Rifaximin boosts the expression of intestinal tight junction proteins and enhances intestinal barrier integrity, preventing the translocation of harmful substances and pathogens. It also inhibits gut dysbiosis caused by influenza virus, reduces conditionally pathogenic bacteria like *E. coli*, and lowers infection risks and disease severity (156).

### 6.2 Probiotics

Probiotics not only prevent intestinal dysbiosis caused by certain diseases and its impact on the intestines, but also exhibit anti-inflammatory effects in the lungs. Research has demonstrated that probiotics have a positive effect on relieving symptoms of influenza and COVID-19 (157). For instance, a study evaluating the anti-influenza activity of four strains of *Lactobacillus plantarum* and two strains of *Bifidobacterium longum* found that treatment with Lp 330, CK10, 920, and Lm 218 reduced the size of viral plaques for rK09 and rAH01 viruses (158). This provides strong evidence for the protective role of probiotics in human viral infections. In hospital intensive care units (ICUs), ventilator-associated pneumonia (VAP) is one of the most prevalent infections among mechanically ventilated patients and is associated with high mortality rates. Nevertheless, randomized controlled trials investigating probiotic treatment for VAP have reported inconsistent results regarding its efficacy (159, 160). Oral administration of probiotics can modulate respiratory immune responses through various signaling pathways. For instance, *Bifidobacterium* promotes Th1/Th2 cell balance and upregulates the secretion of IFN-γ, IL-4, and IL-12 from the spleen (161). *Escherichia coli* reduces recruitment of inflammatory cells in the respiratory tract and suppresses Th2 and Th17 responses (162). *Lactobacillus plantarum* decreases the number of innate immune cells (such as macrophages and neutrophils) as well as cytokine levels (such as IL-6 and TNF-α) in bronchoalveolar lavage fluid (BALF), while inducing immunosuppressive Treg responses in the lungs (163). The intervention effects of different probiotic strains on pulmonary infection via the microbiota-immune axis exhibit significant variation. The underlying mechanisms encompass immune regulation, barrier repair, and metabolic modulation (as detailed in **Table 1**). For critically ill patients, intestinal-targeted therapy necessitates a delicate balance between efficacy and safety. While probiotics may induce bacteremia in immunocompromised individuals (164), such occurrences are typically self-limiting in most cases. However, some probiotics harbor antibiotic resistance genes, such as ARGs (165), which underscores the importance of whole-genome screening to ensure strain safety. Consequently, further extensive studies are warranted to elucidate the potential mechanisms governing the selection of specific probiotic strains, optimal timing, and clinical trials must be carried out to validate their efficacy. Future research should prioritize the precise identification of probiotic strains suitable for treating specific pulmonary infections, the development of more efficient formulations or delivery methods, and the implementation of personalized medicine tailored to individual health statuses and risk factors.

**Table 1:** Differences in the regulation of pulmonary infections by various probiotic strains.

Probiotic Strain	Mechanism of Action	Evidence
*Lactobacillus*	Promotes Th1/Th2 cell balance and upregulates the secretion of IFN-γ, IL-4, and IL-12 in the spleen	*Bifidobacterium* enhances cellular and humoral immune responses, providing protection against influenza infection (161)
*Lactobacillus acidophilus*	Alleviates oxidative stress, reduces NO release, and promotes IL-10 secretion	Administration of *Lactobacillus acidophilus* significantly improves bacterial clearance in the lungs, reduces bacterial load, and prevents bacterial dissemination (166)
*Lactobacillus rhamnosus GG*	Promotes Foxp3 gene expression and modulates Treg cell responses	Modulation of Treg cell responses by *Lactobacillus rhamnosus GG* alleviates pulmonary inflammation and improves outcomes in Pseudomonas aeruginosa pneumonia in mice (167)
*Lactobacillus salivarius FFIG58*	Modulates the release of IFN-β, IFN-γ, IL-6, IL-10, IL-12, and IL-27 from macrophages	*Lactobacillus salivarius FFIG58* provides long-term protection against secondary pneumococcal pneumonia by modulating macrophage responses (168)
*Lactobacillus paracasei*	Stimulates fatty acid-dependent Gpr40/120 to enhance immune responses	Oral administration of *Lactobacillus paracasei MI29* modulates fatty acid metabolism and enhances pulmonary resistance to influenza (169)
*Lactobacillus fermentum*	Inhibits the expression of TLR2/TLR4, reducing the release of IL-4, IL-5, and IL-13	Oral administration of *Lactobacillus fermentum CECT5716* alleviates pulmonary inflammation and gut leak risk in asthmatic mice (170)
*Bifidobacterium breve*	Strongly inhibits neutrophil and eosinophil lung infiltration, reducing the production of IL-1α, IL-1β, and chemokine CXCL2	*Bifidobacterium breve MRx0004* prevents airway inflammation in severe asthma by inhibiting inflammatory responses (171)
*Bifidobacterium lactis*	Upregulates antiviral genes, although changes in gut microbiota are not significant	*Bifidobacterium lactis Bl-04* may influence the host immune response to H1N1 in mice, reducing viral load and improving infection symptoms (172)
*Lactobacillus casei*	Stimulates the release of pro-inflammatory cytokines within the infection site, inducing the formation of polymorphonuclear neutrophil (PMN) clusters	*Lactobacillus casei* enhances non-specific immune activation, strengthening mucosal and systemic immunity to alleviate pulmonary inflammation (173)
*Lactobacillus plantarum*	Activates the STAT5/FOXP3 signaling pathway, increasing Treg cell expression in lung tissue and reducing Th17 cell numbers in peripheral blood, thereby modulating immune responses	*Lactobacillus plantarum* significantly enhances cellular, humoral, and mucosal immunity, alleviating Klebsiella pneumoniae infection (174)
*Escherichia coli*	Reduces the recruitment of inflammatory cells in the respiratory tract and inhibits Th2 and Th17 responses	*Escherichia coli* prevents papain-induced pulmonary inflammation and improves airway hyperresponsiveness (162)

### 6.3 Fecal microbiota transplantation

Fecal Microbiota Transplantation (FMT) is a therapeutic approach that involves transferring the microbial community from the feces of healthy donors into the patient’s intestinal tract via minimally invasive techniques. This process aims to restore the diversity of intestinal microbiota, suppress the proliferation of pathogenic bacteria within the intestinal environment, and establish a competitive exclusion effect against pathogenic bacteria within the local intestinal microbiota. Recent mouse experiments have validated the efficacy of fecal microbiota transplantation (FMT) in ameliorating colonic pathological changes and upregulated TLR expression induced by smoke exposure. Additionally, FMT has been shown to restore intestinal flora diversity and mitigate lung inflammation mediated by IL-17 in models of COPD and smoking-related lung injury. Moreover, fecal microbiota transplantation (FMT) alleviates the cigarette smoke (CS)-induced depletion of blood B cells and spleen CD8+ T cells, as well as the increased circulation of Ly6Clo monocytes. These effects contribute to the restoration of gut microbial balance and the mitigation of lung injury (175). Tang et al. utilized FMT and antibiotic treatment to demonstrate that FMT can enhance the structure of the gut microbiota, promote the production of beneficial metabolites, reduce inflammation, repair the barrier function of alveolar epithelial cells, alleviate lung pathological damage, and significantly improve the prognosis of mice with pneumonia-induced sepsis caused by *Klebsiella pneumoniae*, and FMT restores immune system homeostasis by introducing specific bacterial colonies that produce short-chain fatty acids (e.g., butyrate). Studies involving healthy volunteers have demonstrated that FMT can transiently attenuate the cytotoxicity of systemic immune cells, decrease the proportion of CD8+ T cells and natural killer cells in circulation, while concurrently increasing the proportion of helper T cells (CD4+). This ultimately leads to an elevated CD4+/CD8+ ratio (176). Furthermore, the combination of FMT and antibiotics demonstrates superior efficacy in reducing gut dysbiosis and decreasing the abundance of antibiotic resistance genes (177). Furthermore, FMT restores immune system homeostasis by introducing specific bacterial colonies that produce short-chain fatty acids (e.g., butyrate). Studies involving healthy volunteers have demonstrated that FMT can transiently attenuate the cytotoxicity of systemic immune cells, decrease the proportion of CD8+ T cells and natural killer cells in circulation, while concurrently increasing the proportion of helper T cells (CD4+). This ultimately leads to an elevated CD4+/CD8+ ratio [56]. Nevertheless, its clinical application requires further optimization regarding standardized preparation protocols (e.g., rigorous donor screening) and long-term safety concerns (e.g., potential risks of metabolic disorders).

## 7 Conclusion

As mucosal organs directly exposed to the external environment, the lungs and intestines may harbor similar microbial communities that can modulate local and systemic immune and inflammatory responses. Pulmonary diseases can disrupt intestinal microbial ecology, leading to clinical manifestations of gastrointestinal disorders such as diarrhea and abdominal pain. Moreover, gut-derived metabolites may exacerbate lung inflammation through systemic circulation. Conversely, dysbiosis of gut microbiota could enhance susceptibility to pulmonary diseases and potentially act as causative agents for respiratory infections. Furthermore, antibiotic-induced diarrhea highlights how clinical interventions like antibiotic usage can perturb the balance of gut microbiota, thereby impacting immune defense function and predisposing individuals to lung infections. In the future, alternative intervention methods such as probiotics and fecal microbiota transplantation (FMT) may hold unique potential in the treatment of pulmonary infections. Extensive research has unveiled intricate interactions and mechanisms between the gut and lung infections, encompassing bacterial translocation, short-chain fatty acids, and recognition receptors for pathogen-associated molecular patterns. A comprehensive comprehension of these interaction mechanisms between the gut and lungs is imperative since targeting gut treatments to combat pneumonia could significantly propel medical advancements and enhance global health standards. Therefore, it is crucial to further elucidate the pertinent mechanisms underlying the gut-lung axis. To explore novel avenues for pneumonia treatment, forthcoming experiments involving probiotics/prebiotics should incorporate metagenomic analysis, metabolite studies, assessment of bacterial abundance, as well as analysis of their impact on inflammatory markers from diverse perspectives that influence gut microbiota in experimental research on pneumonia treatment and prevention.

## Glossary

GI: gastrointestinal
COVID-19: Corona Virus Disease 2019
SARS-CoV-2: Severe Acute Respiratory Syndrome Coronavirus-2
CAP: Community-acquired- pneumonia
SCFA: Short-chain fatty acids
ILC: Innate lymphoid cells
Th17: T helper cell 17
Treg: Regulatory T cells
FMT: Fecal microbiota transplantation
GM-CSF: Granulocyte macrophage-colony stimulating factor
NF-κβ: Nuclear Factor kappa-light-chain-enhancer of activated B cells
IFN: Interferon
SFB: segmented filamentous bacteria
H1N1: AH1N1 influenza
VAP: Ventilator associated pneumonia
IFN-γ: Interferon-γ
BALF: Bronchoalveolar lavage fluid
TNF-α: Tumor Necrosis Factor-α
CS: Cigarette Smoke
FOXP3: Transcription factor Forkhead box P3
HDAC: Histone deacetylase
ARDS: acute respiratory distress syndrome
IL-1β: Interleukin-1beta
TJ: tight junction
MLN: Mesenteric Lymph Node
GALT: Gut-Associated Lymphoreticular Tissue
AI-2: Autoinducer 2
MDI: Microbiota Dysbiosis Index
SAP: Stroke-associated pneumonia
TMAO: Trimethylamine N-oxide
GATA3: GATA Binding Protein 3
NOD: Nucleotide-binding oligomerization domain
TUDCA: Tauroursodeoxycholate
AMPK: Adenosine 5’-monophosphate (AMP)-activated protein kinase
AMs: Alveolar Macrophage;DAT, Desaminotyrosine
MX1: Myxovirus Resistance Protein 1
AhR: Aryl Hydrocarbon Receptor
5-HT: 5-hydroxytryptamine
NLRP3: Nucleotide- binding oligomerization domain, leucine- rich repeat and pyrin domain- containing 3
TLR: Toll-like receptors
ROS: Reactive Oxygen Species
CXCL2: C C motif ligand 2
PMN: Polymorphonuclear
ARGs: Antibiotics resistance genes
HPLC: High-performance liquid chromatography
PXR: Pregnane X Receptor
18-HEPE: 18-Hydroxyeicosapentaenoic Acid
GM: Gut Microbiota
iE-DAP: γ-D-Glu-mDAP
RIP2: Receptor-Interacting Protein 2
MDP: Muramyl Dipeptide
IE: indole ethanol
IPA: indole propionic acid
ILA: indole lactic acid
IAA: indole acetic acid
IAld: indole aldehyde
IA: indole acrylic acid
MAT2A: methionine adenosyltransferase 2A
USP16: ubiquitin specific peptidase 16
OVA: ovalbumin
KYN: kynurenine
I3C: Indole-3-carbinol
CLA: Conjugated Linoleic Acid
NEC: necrotizing enterocolitis
BEAS-2B: Human Bronchial Epithelioid Cells
MCP-1: Monocyte chemotactic protein-1
CCL5: chemokine (C-C motif) ligand 5
ICAM-1: Intercellular cell adhesion molecule-1
CA: cholic acid
CDCA: chenodeoxycholic acid
BSH: Bile salt hydrolase
DCA: deoxycholic acid
LCA: lithocholic acid
TGR5: Takeda G protein-coupled receptor 5
ACE2: angiotensin-converting enzyme 2
UDCA: ursodeoxycholic acid
SOD: superoxide dismutase
CAT: catalase
GSH: Glutathione
ALI: acute lung injury
AQP: Aquaporin
LPS: lipopolysaccharide
PPARγ: peroxisome proliferator-activated receptor γ
BMI: body mass index
PTPN2: protein tyrosine phosphatase non-receptor 2
MRSA: methicillin-resistant Staphylococcus aureus
TMAO: Trimethylamine-N-oxide
TMA: Trimethylamine
DMA: dimethylamine
FMO: Flavin MonoOxygenase
PSA: Polysaccharide A
PPDC: phenylpyruvate decarboxylase
CD: Crohn’s disease
IBD: inflammatory bowel disease
PAGln: Phenylacetylglutamine.
